# Nutrient evaluation of a pork by-product and its use as environmental enrichment for managed large exotic cats

**DOI:** 10.1371/journal.pone.0202144

**Published:** 2018-09-12

**Authors:** Cayla J. Iske, Cheryl L. Morris, Jessica D. Colpoys, Kelly L. Kappen, Chelsea A. Iennarella, Anna K. Johnson

**Affiliations:** 1 Department of Animal Science, Iowa State University, Ames, Iowa, United States of America; 2 Omaha’s Henry Doorly Zoo and Aquarium, Omaha, Nebraska, United States of America; University of Illinois, UNITED STATES

## Abstract

North American zoological institutions typically feed ground raw meat diets to large exotic cats. These diets typically are nutritionally complete, but lack physical properties characteristic of whole prey. Lack of mastication and prey manipulation may contribute to behavioral and health challenges. Pork by-products may provide environmental enrichment to mitigate these challenges. The objectives of this study were to evaluate a pig head for nutritional composition and to determine if a pig head was biologically relevant environmental enrichment for managed large exotic cats. Pig heads consisted of: DM: 48.5%; OM: 60.7%; CP: 38.4%; fat: 22.0%; CF: 13.5%; TDF: 3.4%; GE: 4.1 kcal/g DM. Five individually housed exotic cats (*Panthera tigris tigris*, *Panthera tigris altaica* (n = 2), *Panthera tigris jacksoni*, *Panthera leo*) were observed in 2-h blocks, 24-h before pig head introduction (Baseline), at time of pig head introduction (Enrichment) and immediately after the pig head was removed (Post Enrichment) via instantaneous scan sampling for 4 consecutive weeks. Active behaviors were 55.7% higher on Enrichment compared to Baseline days, and 26.4% higher compared to Post Enrichment days (p<0.0001). Active behaviors were 39.8% higher on Post Enrichment compared to Baseline days (p<0.0001). Total active behaviors were highest (p<0.0001) in week 3 and lowest (p<0.0001) in week 4 with differences as high as 64.5% seen among weeks. In conclusion, pig heads have potential to provide nutrient dense enrichment to large exotic cats, and employing a pig head as environmental enrichment increased active behaviors and did not lose novelty.

## Introduction

In North American zoos, a majority of cat diets consist of ground, raw meat, typically horse or beef-based. While these diets have been formulated to meet cat nutrient requirements [1], they do not fulfil other non-nutritive requirements. These may include appetitive behaviors (locating, capturing, and killing prey), feeding process psychology (palatability and physical characteristics) and oral health [2–4]. Carcass-fed cheetahs in zoos have been observed to exercise the use of their feet, teeth, jaws, and head to tear meat from bones, while cats fed raw ground diets do not display these behaviors [5]. Lack of mastication has been identified as a causal factor for misaligned molars that can lead to oral mucosa irritation, resulting in focal palatine erosion [6,7] as well as detrimental alterations in sagittal crest [8], skull growth [9] and skull morphology [10,11].

In the wild, a female cheetah with cubs will spend approximately 40% of her time searching, capturing, and consuming prey [2], and Amur tigers burn nearly 700 kcals daily from hunting [12]. In managed care, much of the searching aspect of hunting is eliminated. Cats may fill this extra time by increasing inactive behaviors [13]. Zoo caregivers have implemented environmental enrichment (EE) devices with the aim of increasing positive species-specific foraging behaviors and increased activity to improve welfare. Environmental enrichment devices or modifications should be biologically relevant to the animal, maintain novelty, and not be detrimental to their health [14,15]. A biologically relevant cat EE device could be food presented in a natural form such as whole prey or whole carcass by-products.

The United States (U.S.) swine industry has potential to provide managed cats in zoos with natural EE in the form of carcass by-products. It has been estimated that by-products comprise 52% of harvested live pig weight [16]. Many by-products sent to rendering, including large bones (femur and humerus) and tails, are extremely nutrient dense and high in cartilage.

In the past three decades, a reluctance to feed raw pork stemmed from concerns associated with *Trichinae* and pseudorabies [17,18]. In the past several years freezing raw pork products, herd biosecurity and vaccination programs, and microbial interventions have aided in reducing these concerns [19–23]. In fact, by 2004 all 50 US states were considered eradicated of pseudorabies, though it persists in some feral hogs [24]. Only 90 cases of *Trichinae* were reported by the Centers for Disease Control (CDC) between 2008 and 2012 and of those only 24% of cases were due to raw pork [25] and freezing at –20°C for 8 minutes or for 64 minutes at –15°C will kill *Trichinae*, indicating these two concerns are very low risk from pork sourced from US swine operations and frozen prior to feeding [26]. Additionally, recent studies feeding commercially available raw pork diets have found no clinical symptoms when fed to exotic cats [27,28].

Pig heads are one of the largest by-products in size and weight. It is hypothesized that pig heads would be highly-valued EE, requiring manipulation and mastication by managed cats in zoos. Therefore, the objectives of this experiment were to: 1) determine if a pig head could be a biologically relevant EE device for large cats managed in zoological settings and 2) to evaluate pig heads for chemical composition for nutrient intake calculations.

## Materials and methods

Animal care and husbandry protocols were approved for the current study by Omaha’s Henry Doorly Zoo and Aquarium’s (Omaha) Institutional Animal Care and Use Committee (IACUC) and Iowa State University’s Institutional Animal Care and Use Committee (IACUC).

### Animals and diet

Five exotic cats were used; four located at Omaha’s Henry Doorly Zoo and Aquarium, Omaha, Nebraska (Omaha) and one at Blank Park Zoo in Des Moines, Iowa (Blank Park) (Table 1). Cats were housed individually and fed their normal amount and type of raw meat diet, without fasting days, and all were cared for by zoo caregivers (Table 2). Cats had previously been offered whole prey intermittently, but was not part of their daily diet during the current study. The experiment was conducted from June 30 to July 23, 2014.

**Table 1:** Description of cats (n = 5) observed before (baseline; normal zoo enrichment devices), during (enrichment; pig head), and after (post enrichment; normal zoo enrichment devices) provision of pig head for four consecutive weeks housed at Omaha’s Henry Doorly Zoo and Aquarium and Blank Park Zoo from June 30 to July 23, 2014.

Common name	Scientific name	Sex	Age (years)	Weight (Kg)	Time at current facility (months)
**Omaha**					
Bengal Tiger	*Panthera tigris tigris*	Female	9	99.8	91
Amur Tiger	*Panthera tigris altaica*	Female	9	105.3	31
Malayan Tiger	*Panthera tigris jacksoni*	Female	17	75.3	126
African Lion	*Panthera leo*	Male	15	245.4	150
**Blank Park**					
Amur Tiger	*Panthera tigris altaica*	Female	17	125.6	182

**Table 2:** Housing specifications of cats (n = 5) observed before (baseline; normal zoo enrichment devices), during (enrichment; pig head), and after (post enrichment; normal zoo enrichment devices) provision of pig head for four consecutive weeks at Omaha’s Henry Doorly Zoo and Aquarium and Blank Park Zoo from June 30 to July 23, 2014.

Common name	Space (m^2^)	Water^a^	Feed	Raw diet fed daily (Kg)	Indoor/Outdoor Access^b^	Exhibit Style
**Omaha**						
Bengal Tiger	151.4	Pool	Morning, on exhibit^c^	4.7	Outdoor	Natural^e^
Siberian Tiger	49.2	Bowl	Morning, on exhibit	4.9	Indoor	Cement/brick
Malayan Tiger	111.5	Bowl	Morning, on exhibit	2.3	Indoor and Outdoor	Cement/brick
African Lion	124.5	Bowl	Morning, on exhibit	4.7	Indoor and Outdoor	Cement/brick
**Blank park**						
Siberian Tiger	464.5	Pool	Evening, in back holding^d^	3.9	Outdoor	Natural

^a^ Electronic water bowls: Model 760-10W, Nelson Manufacturing Company, Cedar Rapids, IA.

^b^ Access was constant for each cat and never changed throughout the study.

^c^ Between 0730 and 0900-h in publically viewable enclosure.

^d^ Between 1700 and 1800-h out of public view.

^e^ Dirt and grass flooring with logs, rocks, trees, and natural stream/pool in enclosure.

### Treatments and experimental design

The enclosure containing each cat was the experimental unit. The experimental design was a complete randomized design. Cat behavior was evaluated for three consecutive days each week for four consecutive weeks. There were three treatment days; ***Baseline*** (24 hr prior to the environmental enrichment device being placed into the housing enclosure but cats had access to normal zoo enrichment devices), ***Enrichment*** (the cat had access to the pig head environmental enrichment device in the housing enclosure but not the normal zoo enrichment devices) and ***Post Enrichment*** (after the pig head environmental enrichment device was removed from the housing enclosure but cats had access to normal zoo enrichment devices). Weeks consists of one Baseline (normal zoo enrichment devices), one Enrichment (pig head), and one Post Enrichment (normal zoo enrichment devices) day.

Pig heads were offered at same time as standard diet and normal zoo enrichment devices were removed on Enrichment days to evaluate behaviors and use of the pig head directly compared to normal devices.

### Environmental enrichment device

No cats in this study had previously been exposed to a pig head based on records and zoo caregiver interviews. Heads were provided by Sustainable Swine Resources, LLC, Sheboygan Falls, WI) and were shipped, frozen, to Omaha and stored frozen at -18°C until 24 hr prior to offering, at which time they were moved to a cooler (2°C). Heads offered to the tiger at Blank Park Zoo were transported, frozen to Des Moines and handled in the same way. Each head still contained the brain, eyes, teeth, bone, as well as some muscle and fat. The lower jaw and snout had been removed during processing.

On Baseline and Post Enrichment days, normal zoo EE devices included a maximum of two items daily and could include a combination of plastic balls, plastic cylinders, plastic logs, plastic crates, and newspaper which were offered evenly between cats. No scents or food items were used. Pig heads were removed from the exhibit between 0730 and 0900 on Post Enrichment days, 24 hr after initial offering, and weighed immediately.

**Fig 1:**
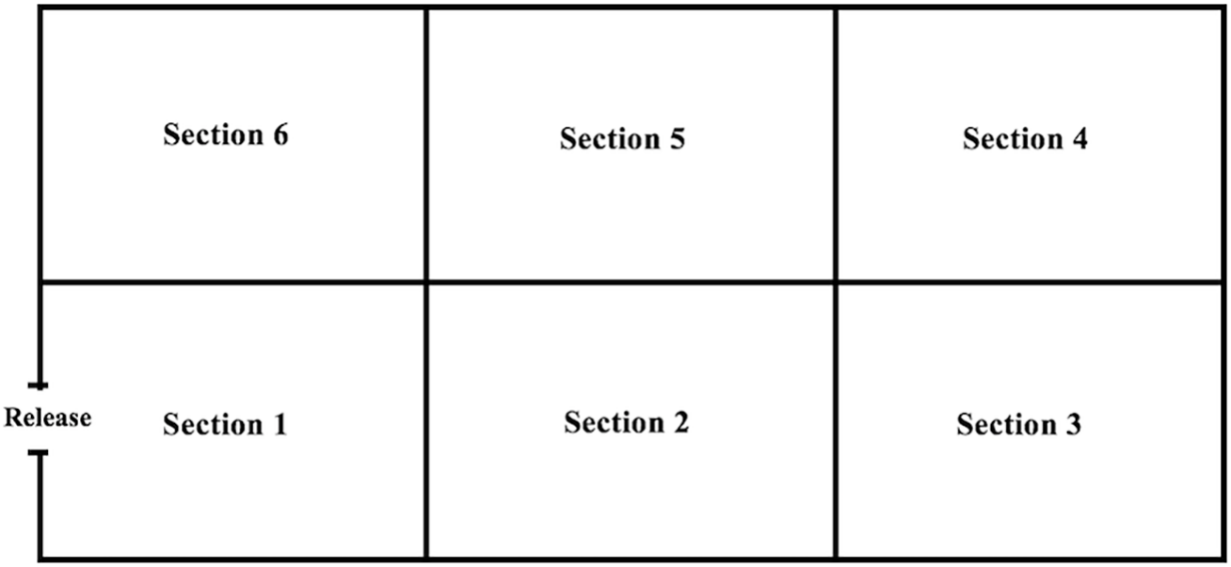
Section enclosure locations for large exotic cats housed at Omaha’s Henry Doorly Zoo and Aquarium and Blank Park Zoo.

Each enclosure was divided into 6 equal location sections. Initially each enclosure was divided in latitudinal halves and then each half into three equal sections longitudinally. For ease of tracking during observation, landmarks (logs, bricks, trees, rocks, etc.) already present in the enclosure were used to distinguish one section from another. Cats were released from section 1 (defined as the first section that each cat had access to upon release from back holding quarters; (Fig 1)). All enrichment devices were placed in section 3 and diet was placed in section 4, so that diet and enrichment were equidistance from the cat when it entered the enclosure. The exceptions to this were the Bengal tiger housed at Omaha that received diet in section 1 and enrichment in section 6 because it was fed in a cove that could not be moved and the Amur tiger house in Des Moines that was fed off exhibit.

### Behaviors

Cats were released from back-holding quarters each morning between 0730 and 0900. One min instantaneous scan sample intervals were used to record behavior via live observations [29]. Observation timing began when the cat’s nose broke the door barrier upon release from back-holding quarters and the first scan was obtained one min later and recording continued for 120 min (Table 3). The ethogram was adapted from Skibiel et al., 2007 and Wells and Egli, 2004 [30,31]. Time to approach the pig head began when the cat’s nose broke the door barrier and ended when the cat first investigated the pig head. Enclosure section locations were observed using 5 min interval samples. A cat’s nose had to be inside the location for it to be noted in that location section.

**Table 3:** Ethogram used for live observations of large cat behaviors via instantaneous scan sampling before (Baseline; normal zoo enrichment devices), during (enrichment; pig head), and after (post enrichment; normal zoo enrichment devices) provision of pig head for four consecutive weeks at Omaha’s Henry Doorly Zoo and Aquarium and Blank Park Zoo from June 30 to July 23, 2014.

Measure^a^	Definition
**Active**	
Locomotion	Walking, running, climbing, pacing, jumping in a non-investigatory manner (head above shoulders)
Standing	All four feet touching the ground and body held in upright posture
Rolling	Laying on one side and completely rotating to the other side
Exploring	Sniffing ground or enclosure features in an investigatory manner (head below shoulders to ground), scratching, licking, or sniffing any part of enclosure
Grooming	Directing licking or scratching to own body
Head over water	Head hovering over water or drinking water
Vocalizing	Making any noise coming from mouth
Interest in item	Oriented (in same section) towards EE item; sniffing or walking towards, but not touching
Interaction with item	Any part of the cat is physically touching the enrichment item
Interest in diet	Oriented (in same section) towards diet; sniffing or walking towards, but not touching
Interaction with diet	Any part of the cat is physically touching the diet
Spraying^b^	Spraying from the posterior for the purpose of scent marking (not urination)
**Inactive**	
Laying	Laying down and immobile
Sitting	Front legs extended and back legs bent with posterior on ground
**Other**	
Eliminatory	Any projection of bodily fluids i.e. urination, defecation
Unobserved	Observer could not see the cat

^a^ All behaviors were mutually exclusive per scan.

^b^ Spraying was distinguished from eliminatory behaviors (which was defined as regular urination and defecation) as being spraying of objects for scent marking purposes, not steady urination.

A total of 6 observers observed the cats. One trainer with previous large cat behavioral experience was responsible for training prior to study commencement as described by Caro et al. (1979) [32] and Martin and Bateson (1993) [33]. Inter-observer reliability testing occurred at Omaha or the Animal Behavior and Well-Being laboratory at Iowa State University. The trainer reviewed and scored 10-min videos of one jaguar (*Panthera onca*), one puma (*Puma concolor*), and one African lion (*Panthera leo*) from the Omaha zoo using the ethogram (Table 3) and a one min scan sampling interval [29]. Quantification of stereotypic behaviors was not an intent of this study and are not included in the ethogram. The five trainee observers independently reviewed and scored the same three videos using the same recording rule. Inter-observer agreement was ≥ 93% [34].

### Temperature and relative humidity

Temperature and relative humidity (RH) were recorded at each study location every 10 min during the study using HOBO sensors (HOBO Model H08-003-02, Onset Computer Corp., Bourne, MA). One sensor was placed outdoors in the shade, under an awning, approximately 5 m from the ground. The other sensor was placed in the indoor area of one enclosure, on a window cutout, approximately 4 m from the ground. Data were transferred to a computer using HoboWareLite (Onset Computer Corp., Bourne, MA). Data were averaged for the two hr the cats were observed, using averages from the sensor(s) where the cat had access (indoor, outdoor, or both).

### Orts and fecal scores

Cats received the same amounts of their respective raw meat diets during the study as prior to study initiation. Unconsumed diets (orts) were collected, weighed, and recorded daily by zoo caregivers or a trained study observer. Orts were recorded for each observation day the morning following observations (i.e. Baseline observations took place on Monday morning with orts and fecal scores recorded on Tuesday morning). Weekly orts were calculated as average Baseline, Enrichment, and Post Enrichment day orts within each week. Percent orts were calculated by dividing grams of orts by total diet offered (wet weight) and dividing by 100. Diet orts were weighed to the nearest 0.1g (Mettler Toledo XP8001M Precision Balance, Columbus, OH). Fecal scoring was conducted daily when orts were collected and were visually assessed on a five-point scale of: 1 = hard, dry pellets; 2 = dry, well-formed; 3 = soft, moist, formed; 4 = soft, unformed; 5 = watery liquid [35].

### Head weight loss

Each head was individually weighed to the nearest 0.1g (Mettler Toledo XP8001M Precision Balance, Columbus, OH) 24 hr prior to placement in the housing enclosure. The head was placed in the housing enclosure on the Enrichment morning and approximately 24 hr later the head was removed and re-weighed using the same scale. The difference between initial and ending head weight was recorded as head weight loss (g). Intrinsic head weight loss (g) was measured to determine weight loss from thawing and dehydrating in the laboratory. This was done by weighing three heads (3.0, 3.5, and 4.0 kg in weight) while frozen, and re-weighing after 24 hr when housed, untouched, at 24° C. Difference between each starting and finishing weight was defined as intrinsic head weight loss and was used to calculate intrinsic loss of each pig head used in the study.

### Pig head analyses

Three pig heads were collectively passed twice through a mechanical grinder (Buffalo No. 66BX Enterprise, St. Louis, MO), and then passed twice through a Hobart 52 grinder with a 5-mm die (model number 4046; Hobart Corporation, Troy, OH) for homogenization. Entire pooled samples were then frozen and freeze dried for 4 days (vacuum/freezer: Uni-Trap by Cenco Model #10–100; vacuum chamber: Virtis Model #10-104-LD). Grinding and freeze drying were conducted at Iowa State University. After freeze drying, samples were ground through a 2-mm screen (Wiley mill, model 3383-L10, Thomas Scientific, Swedesboro, NJ) and analyzed for chemical composition. Heads were analyzed for dry matter (DM) (Method 934.01) and organic matter (OM ((Method 942.05) [36]). Crude protein (CP) was determined using a Leco Nitrogen/Protein Determinator (Method 992.15) (model FP-528, Leco Corporation, St. Joseph, MI). Fat concentrations were determined by hexane extraction ((Method 991.36) [37]). Protein to fat ratios were calculated by dividing protein concentration by fat concentration. Gross energy (GE) was determined by bomb calorimetry (model AC 500, Leco Corporation, St. Joseph, MI). Crude fiber (CF) was determined by Midwest Laboratories ((Omaha, NE) (AOCS Ba6a-05 [38])). Total dietary fiber (TDF) was also determined [39] using triple the amount of protease and double the time for the water bath after addition of the protease, for a high protein sample. Metabolizable energy (ME) of the head was calculated using unmodified Atwater values (9.0 kcal/g fat, 4.0 kcal/g protein, 4.0 kcal/g carbohydrate) multiplied by fat, protein, and carbohydrate concentration of each diet [1]. Carbohydrate concentrations of diets were calculated by difference as nitrogen free extract (NFE) using the following equation (DMB): (100 –(% ash + % CP + % fat + % TDF). Though crude fiber is typically used in this calculation, TDF is a more accurate measure of dietary fiber; therefore, TDF was used for calculating NFE [40,41]. Due to expected very low fiber levels and the cumulative nature of the calculation, NFE of some items produced a negative number, in which case a value of zero was used for NFE. Mineral analyses were conducted by Midwest Laboratories (Omaha, NE; Method 985.01) [42]; (MWL ME PROC 29)]. Chemical analyses were conducted in the Nutrition laboratory at Omaha’s Henry Doorly Zoo and Aquarium unless otherwise noted.

### Meat to bone and fat to bone ratios

Meat-to-bone and fat-to-bone ratios were determined by manually separating meat and fat from the skull bone of one 3.5 kg skull using hand-held knives. Meat, fat, and skull (including brain) components were then weighed separately to the nearest 0.1 g (Mettler Toledo XP8001M Precision Balance, Columbus, OH) and ratios were calculated.

### Nutrient intakes from pig heads

Nutrient intakes by cats, from head consumption only, were calculated based on macronutrient composition of head, head weight loss, and intrinsic losses. This was done via the following equation: actual intake = (head weight loss (g))—(starting head weight (g) * % intrinsic loss). Macronutrient and energy concentrations (dry matter basis) were then multiplied by dry matter intake (g) to yield intake of macronutrients (g) and energy (kcal) (dry matter basis) from consuming the head.

### Statistical analysis

All data were evaluated for a normal distribution before analysis by using the UNIVARIATE procedure of SAS (SAS Institute Inc., Cary, NC). Data that failed to meet the assumption of normally distribution (active, inactive and other behaviors and postures, location change, time to approach head and orts) were analyzed using the GLIMMIX procedure of SAS and default significant convergence had to be relaxed from 1x10^-8^ to 0.0001. All models included the fixed effect of treatment (week, day and week*day interaction), random effect of cat, and tested temperature, RH, and enclosure as covariates. Insignificant variables were removed from the final model. A binomial distribution was used in evaluation of active, inactive, and other behaviors and postures while Poisson distribution was utilized in the evaluation of location changes and gamma distribution was used in the evaluation of head weight loss and time to approach. Data used to evaluate fecal scores met the assumption of normal distribution and were analyzed using the MIXED procedure of SAS. A p value of ≤ 0.05 was considered significant. Mutually exclusive individual behaviors could not be analyzed statistically due to a small sample size and few/variable numbers of observations of those behaviors; therefore, they are presented descriptively. Chemical and mineral composition of various pork-based enrichment items, orts, and minimum and maximum time to approach the head are presented descriptively. Separating age, sex, and species effects was not an intention of this study.

## Results

### Behaviors

Temperature and enclosure size were not significant covariates in any measures of behavior and RH was significant (P = 0.003) as a quadratic covariate only in total active and inactive behaviors. On Enrichment days, active behaviors were 55.7% higher compared to baseline and 26.4% higher compared to Post Enrichment days respectively (p<0.0001). On Post Enrichment days, active behaviors were 39.8% higher than Baseline days (p<0.0001; Table 4). Active behaviors were highest in week 3 and were 44.3, 49.5, and 64.3% higher compared to weeks 1, 2, and 4, respectively (p<0.0001; Table 5).

**Table 4:** LSMeans (±SE) percentage of time for a 2-h period for large exotic cats engaged in active^d^, inactive^e^, and other^f^ behaviors and postures before (Baseline; normal zoo enrichment devices), during (Enrichment; pig head), and after (Post Enrichment; normal zoo enrichment devices) provision of a pig head over 4 consecutive weeks housed at Omaha’s Henry Doorly Zoo and Aquarium and Blank Park Zoo from June 30 to July 23, 2014.

Behavior	Baseline	Enrichment	Post Enrichment
**Active**	22.6 ± 5.9^a^	51.1 ± 8.2^b^	37.6 ± 7.8^c^
**Inactive**	76.8 ± 6.0^a^	48.9 ± 8.2^b^	62.4 ± 7.7^c^
**Other**	0.7 ± 0.001	0	0

^a-c^ Means within a row lacking a common superscript letter are statistically different at p<0.0001.

^d^ Locomotion, standing, rolling, exploring pen, grooming, head over water source, vocalizing, interest in item, interaction with item, interaction with diet, interest in diet, and spraying.

^e^ Laying and sitting.

^f^ Eliminatory and unobserved.

**Table 5:** LSMeans (±SE) percentage of time for a 2-h period for five large exotic cats spent engaged in active^d^, inactive^e^, and other^f^ behaviors and postures during provision of a pig head for four consecutive weeks^g^ housed at Omaha’s Henry Doorly Zoo and Aquarium and Blank Park Zoo from June 30 to July 23, 2014.

	Week			
Behavior	1	2	3	4
**Active**	34.2 ± 7.5^a^	31.0 ± 7.1^a^	61.0 ± 8.1^b^	21.9 ± 5.7^c^
**Inactive**	65.2 ± 7.5^a^	68.5 ± 7.1^a^	39.0 ± 8.1^b^	77.6 ± 5.8^c^
**Other**	0.6 ± 0.001	0.5 ± 0.002	0	0.5 ± 0.001

^a-c^ Means within a row lacking a common superscript letter are statistically different at p<0.0001.

^d^ Locomotion, standing, rolling, exploring pen, grooming, head over water source, vocalizing, interest in item, interaction with item, interaction with diet, interest in diet, and spraying.

^e^ Laying and sitting.

^f^ Eliminatory and unobserved.

^g^ Weeks consists of one Baseline (normal zoo enrichment devices), one Enrichment (pig head), and one Post Enrichment (normal zoo enrichment devices) day.

Descriptively, locomotion and laying were the most frequently observed postures across all treatment days and weeks (approximately 80% of the total average time). Lying was 22.1% higher on Baseline compared to Enrichment days and sitting was 64.1% higher on Baseline compared to both Enrichment and Post Enrichment days (Table 6).

**Table 6:** Descriptive average percentage of observation block time for a 2-h period for five large exotic cats spent engaged in mutually exclusive behaviors and postures before (baseline [B]; normal zoo enrichment devices), during (enrichment [E]; pig head), and after (post enrichment [P]; normal zoo enrichment devices) provision of a pig head for four consecutive weeks housed at Omaha’s Henry Doorly Zoo and Aquarium and Blank Park Zoo from June 30 to July 23, 2014.

	Day, %			Week, %				
Measure	B	E	P	1	2	3	4	SD
**Active**								
Locomotion	19.61	19.51	23.74	21.98	16.47	30.99	15.22	20.12
Standing	4.23	3.12	3.21	4.02	3.09	3.65	3.31	2.44
Rolling	0.05	0.00	0.00	0.00	0.06	0.00	0.00	0.11
Exploring	1.42	0.87	1.33	1.38	1.10	1.58	0.76	1.62
Grooming	4.78	4.32	3.08	3.92	3.53	4.75	4.20	3.88
Head over water	0.51	1.01	0.51	0.88	0.83	0.34	0.55	0.85
Vocalizing	0.83	0.46	0.60	0.33	0.66	0.76	0.83	1.00
Interest in item^a^	0.09	0.09	0.09	0.11	0.00	0.07	0.21	0.26
Interaction with item^a^	0.05	17.95	0.18	5.73	4.57	7.78	6.61	14.02
Interest in diet	0.12	0.00	0.30	0.21	0.07	0.28	0.00	0.43
Interaction with diet	3.42	2.18	3.36	2.82	2.96	3.03	3.21	2.65
Spraying	0.00	0.05	0.00	0.00	0.06	0.00	0.00	0.11
**Inactive**								
Laying	64.54	50.28	63.73	58.83	66.39	46.63	64.67	20.87
Sitting	0.78	0.28	0.28	0.17	0.28	0.55	0.90	1.07
**Other**								
Eliminatory	0.28	0.23	0.37	0.17	0.44	0.28	0.28	0.68
Unobserved	0.09	0.14	0.05	0.06	0.11	0.14	0.07	0.31

^a^ Enrichment item on Baseline (B) and Post Enrichment (P) days was zoo’s environmental enrichment items provided per standard operating procedure; on Enrichment (E) days the enrichment item was the head. Interest was defined as oriented towards, but not touching while interaction was defined as cat physically touching the object.

Interaction with EE devices differed by day. All cats were seen interacting with the pig head on Enrichment days. Descriptively, on Enrichment day, cats interacted with the pig head approximately 98% more compared to Baseline and Post Enrichment days when the cats were offered the typical zoo enrichment items. Interaction with typical zoo enrichment items was 72.2% higher on Post Enrichment compared to Baseline days. Interaction with the pig head did not decrease over the four-week study, with more pig head interaction occurring in weeks 3 and 4 (7.8 and 6.6%, respectively) than in weeks 1 and 2 (5.7 and 4.6%, respectively; Table 6).

Average time to approach the pig head (±SE) did not differ over the four weeks (p = 0.45; 74.7±50.3; 82.0±55.2; 38.0±27.0 and 36.9±26.3 sec for Weeks 1–4, respectively). Descriptively, minimum time to approach the head was ~9 sec, with a maximum time being ~292 sec. There were no differences in number of location section changes between Baseline, Enrichment, or Post Enrichment days (p = 0.67). Number of location section changes was greater (P<0.05) in week 3 (8.8±1.9) compared to weeks 2 (6.7±1.5) and 4 (6.0 ± 1.4) but was not statistically different (P = 0.14) from week 1 (7.2±1.6).

### Temperature and relative humidity

Average temperatures (±SD) were 26.0±1.4, 24.4±3.3, and 22.6±2.6°C for Baseline, Enrichment, and Post Enrichment days, respectively, and 23.0±2.9, 25.1±1.8, 22.3±2.0, and 27.1±2.0°C for weeks 1, 2, 3, and 4, respectively. Average RH (±SD) averaged 71.4±10.3, 62.2±8.1, and 61.0±4.1% on Baseline, Enrichment, and Post Enrichment days, respectively, and 68.6±11.2, 63.6±5.9, 57.6±4.3, and 69.1±8.7% for weeks 1, 2, 3, and 4, respectively.

### Orts and fecal scores

Orts data could not be analyzed statistically because of the small sample size (n = 5) but numerically averaged (±SD) 325.2±720.2, 279.8±542.2, and 23.2±53.7 g (or 7.0±14.8, 8.6±17.4, and 0.5±1.1% of offered diet) for Baseline, Enrichment, and Post Enrichment days, respectively, and 274.8±547.7, 179.6±593.4, 17.4±41.4, and 357.0±666.5 g (or 8.3±17.7, 3.7±12.1, 0.5±1.2, and 8.6±15.0% of offered diet) for weeks 1, 2, 3, and 4, respectively. Temperature, RH, and enclosure size were not significant covariates in fecal score analysis. Fecal scores did not differ over treatment days (Baseline: 3.6±0.2; Enrichment: 3.7±0.2; Post Enrichment: 3.7±0.2; p = 0.77) or over the four weeks (3.6±0.2, 3.6±0.2, 3.8±0.2, 3.6±0.2, for weeks 1–4, respectively; p = 0.68). No health issues, defined as excessively loose stools, vomiting, or abnormalities documented by keepers, were noted during the duration of the experiment.

### Head weight loss

Temperature, RH, and enclosure size were not significant covariates in head weight loss. Starting weights ranged from 2776.0 to 4495.1 g. Head weight loss (g) (±SE) did not differ over the four weeks of study (543.4±220.9, 587.6±220.9, 555.1±223.9, and 700.3±223.9, respectively; p = 0.37). Starting and ending weights of heads used to determine intrinsic weight loss averaged 3530.5 and 3450.7 g, respectively, indicating an average intrinsic weight loss of 2.3% in 24 hr.

### Pig head analyses

Nutrient composition of analyzed heads is presented in Table 7. Pig heads contained 48.1% dry matter, and 38.4, 22.0, 13.5, and 3.4% CP, fat, CF, and TDF, respectively, on a dry matter basis. The gross and metabolizable energy concentration was 4.1 and 3.5 kcal/g DM, respectively. Calcium and phosphorus concentrations was 13.7 and 6.6% DM, respectively, indicating a calcium to phosphorus ratio of 2:1.

**Table 7:** Chemical composition of pig heads (DM basis)^a^^,^^b^.

		DM Basis
Unit	Nutrient, DM basis	Concentration
%	DM	48.5
	OM	60.7
	CP	38.4
	Fat	22.0
	CF	13.5
	TDF	3.4
	Protein:Fat Ratio^c^	1.8
Kcal/g	GE	4.1
	ME^d^	3.5
	Mineral	
Total %	Ca	13.7
	P	6.6
	S	0.3
	K	0.3
	Mg	0.3
	Na	0.6
	Fe	171.1
	Ca:P Ratio^e^	2.1
PPM	Mn	2.1
	Cu	3.9
	Zn	117.1
Ratios^f^		
Meat:Bone	0.3	
Fat:Bone	0.1	

^a^ Three pig heads were homogenized and analyzed to give average composition.

^b^ Abbreviations used: DM, Dry matter; OM, organic matter; CP, crude protein; CF, crude fiber; TDF, total dietary fiber; GE, gross energy; Ca, calcium; P, phosphorus; S, sulfur; K, potassium; Mg, magnesium; Na, sodium; Fe, iron; Mn, manganese; Cu, copper; Zn, zinc; PPM, parts per million.

^c^ Protein:fat ratios were calculated by dividing protein concentration by fat concentration.

^d^ ME = Calculated using Atwater values: 9 kcal of ME/g of fat + 4 kcal of ME/g of CP + 4 kcal of ME/g of N-free extract.

^e^ Ca:P ratios were calculated by dividing calcium concentration by phosphorus concentration.

^f^ Meat:bone and fat:bone ratios were determined by manually separating meat and fat from skull bone using hand-held knifes. Meat, fat, and skull (including brain) components were then weighed separately to the nearest 0.1 g.

### Meat to bone and fat to bone ratios

There were 338.5 g of fat and 658.0 g of meat (including eyes) removed from the skull. Determination of fat and meat was made by the person separating the components, with white substance separated as fat and red being meat. The ending weight of the skull (with brain still included) was 2343.9 g. The estimated meat to bone ratio of the head was 0.3:1 and the fat to bone ratio was 0.1:1, with the brain remaining in the skull (Table 7).

### Nutrient intakes from pig heads

On average 266.1 g of dry matter from pig heads was consumed daily consisting of 161.5, 102.2, 58.6, and 9.2 g OM, CP, fat, and fiber (TDF), respectively. Gross and metabolizable energy average intakes were 1088.2 and 939.2 kcal/d, respectively. Minimum and maximum dry matter intakes ranged from 37.0 to 647.0 g/d. Crude protein, fat, and fiber intakes ranged from 14.2 to 258.9 g, 8.1 to 148.4 g, and 1.3 to 23.2 g, respectively. Likewise, GE intakes were variable and ranged from 151.3 to 2756.8 and ME ranged from 130.6 to 2379.4 kcals (Table 8).

**Table 8:** Macronutrient intake from pig head of cats (n = 5) after being offered a pig head at Omaha’s Henry Doorly Zoo and Aquarium and Blank Park Zoo from June 30 to July 23, 2014.

	g (DMB)							kcals	
	Apparent Intake^a^	Actual Intake^b^	DM	OM	CP	Fat	TDF	GE	ME
**Average**	630.3	548.5	266.1	161.5	102.2	58.6	9.2	1088.2	939.2
**Minimum**	154.4	76.3	37.0	22.5	14.2	8.1	1.3	151.3	130.6
**Maximum**	1478.5	1389.5	647.0	409.1	258.9	148.4	23.2	2756.8	2379.4

^a^ Apparent intake of the head was determined by calculating difference between weight of head while frozen, before offering, and 24-h after offering to cats.

^b^ Actual grams of intake were calculated as follows: actual intake = (head weight loss (g))—(starting head weight (g) * % intrinsic loss). Intrinsic losses for heads weighing ~3 kg = 2.12%, ~3.5 kg = 2.32%, ~4 kg = 2.31%. Macronutrient intakes were calculated by multiplying head macronutrient composition by actual intake amounts (g).

## Discussion

Visitor education, public perception [43] and Association of Zoos and Aquariums accreditation requirements [44] have resulted in increased utilization of zoo environmental enrichment over recent decades. While many creative and unique environmental enrichment devices have been developed for managed, large exotic cats, relatively little is known about how these tools impact cats both behaviorally and nutritionally.

### Behaviors

Enrichment that considers the natural history and inherent interest and nature of an animal is said to be biologically relevant [45,46]. Offering biologically relevant enrichment may stimulate species-specific behavior [47,48] reduce stress [49,50] and maintain or improve welfare. A pig head must be manipulated and masticated to be consumed and is in a form that might be found by a cat in the wild, making it a biologically relevant EE. Consumption of a pig head requires time and effort; therefore, may alter the cat’s activity budget.

In this study, on Enrichment days, cats engaged in more active behaviors and postures suggesting that offering a pig head as enrichment was successful at increasing species-specific behaviors compared to the typical enrichment devices provided by the zoo on Baseline- and Post Enrichment days. Furthermore, this increased activity continued into Post Enrichment, suggesting that the pig head had a sustained, and positive effect on the large cat behavioral repertoire. Additionally, enrichment that provides behavioral effects after removal is valuable both financially and from a management standpoint with fewer enrichment items needing to be purchased and offered. It should be stated, however, that the current study did not assess for stereotypic behaviors directly and this should be assessed in future studies to determine influence of pig head provision on stereotypies.

Our study agrees with previous work that offered a horse knuckle or beef shank to tigers, ocelots, cougars, cheetahs, and lions. In this work, animals increased their active behaviors by nearly 50% compared to no enrichment [30]. Offering bones to African lions twice per week resulted in increased (nonstereotypic) activity by more than 66% compared to no enrichment [51]. Bashaw and colleagues (2003) also observed a sustained activity increase two days after bones were given, with activity being 40% higher than no enrichment. Therefore, further work is necessary to assess if increased cat activity is sustained more than 24 hr after offering of a pig head.

In the current study, highest total active behaviors and postures observed on Enrichment days likely resulted from a 99.0% increase in pig head interaction compared to Baseline- and Post Enrichment days that offered typical zoo environmental enrichment devices. Higher Post Enrichment activity compared to Baseline days could likely be attributed to more observations of locomotion (17.4% increase), interaction with normal zoo enrichment devices (72.2% increase), and diet interest (60.0% increase). In the current study, objectives were to evaluate differences in overall active and inactive behaviors as this is the first study to evaluate the use of a pig head as EE. Therefore, stereotypic behaviors such as pacing were not under direct evaluation, however, should be a consideration for subsequent studies. It is interesting to note that interaction with normal zoo enrichment devices was increased after the pig head offering (between Baseline and Post Enrichment days). Interaction with the pig head enrichment device did not decrease across the four weeks of this study suggesting that these cats did not habituate to the pig head when it was offered once a week. Skibiel and colleagues (2007) [30] monitored behaviors for 7 days following provision of bones, frozen fish, and spices and determined that increased activity in large exotic cats was not sustained after enrichment offering. Therefore, future work should extend the timeframe that a pig head is offered to determine if there is a point where habituation occurs in cats.

Decreased activity during week 4 was likely due to temperature as, on average, temperature was 4.8°C higher in week 4 (27.1°C) compared to other weeks (23.0, 25.1, and 22.3°C in weeks 1, 2, and 3, respectively). Additionally, highest activity was seen in week 3 in which average temperatures were 2.8°C lower. Future work should extend this study over all seasons to see how temperature and/or RH directly affect cat activity and interaction with the pig head as these variables cannot be ruled out as influencing factors.

Results of this study are valuable for animal managers in zoological institutions that aim to increase large cat activity through environmental enrichment whilst also providing nutritional diversity. More active large cats have also been shown to increase zoo visitor interest and attention [52]. Similar research is warranted in other carnivore species to determine if results are similar across species and with the use of various pork by-products as enrichment items. In addition, further research with these enrichment items may also be conducted to directly address their effects on stereotypies in animals known to display them and effect of varying predictability (placement and timing of offering).

### Orts and fecal scores

Giving a pig head as an enrichment device did not result in a detrimental effect on raw diet intake. In fact, fewer orts on Enrichment and Post Enrichment days compared to Baseline indicated higher consumption. More orts in week 4 and fewest orts in week 3 may be explained by the fluctuation in temperatures during those weeks (highest average temperature on week 4 and lowest on week 3). This may warrant further investigation, perhaps across seasons, as few previous studies have evaluated standard diet consumption with the offering of dietary enrichment. In addition, giving the pig head did not appear to negatively affect the overall intestinal health and digestive function of the cats as fecal scores ranged from 3.6 to 3.8 (3 being ideal).

### Head weight loss

Loss of head weight from cat consumption remained similar over the study and provides further evidence that no habituation occurred. Intrinsic weight loss was determined to differentiate inherent loss from loss associated with actual consumption by the cat. For example, if a 4.3 kg head only lost 100 g after being offered to a cat, it is likely due to intrinsic losses. However, if the head lost 800 g, the cat likely consumed 700 g of the head weight. This can then be used to adjust the animal’s typical diet to ensure the proper caloric and nutrient intake. However, further studies should consider intrinsic losses at varying temperatures.

### Pig head analyses

The meat to bone (0.3) and fat to bone (0.1) ratios of the pig head indicated the majority of head was skull bone and a substantial portion of the head could be manipulated and consumed in a 24-hr period providing additional daily calories from fat and protein above the normal diet consumed. In the current study, none of the cats fully consumed the head, nor did they reach the brain cavity. Ratios of meat and fat to bone should also be determined in pig heads of various weights in future studies to assess ratio differences in head size. Future work should also evaluate nutrient composition of individual pig head components such as meat, fat, bone, eyes, and brain.

Potential consumption of enrichment items that could alter overall nutrient intake of an animal requires evaluation. The Ca:P ratio of pig heads was 2.1:1, which is close to suggested ideal intakes for cats (2:1) [53]. Therefore, consumption of the head should not alter the calcium to phosphorus balance of the overall diet; however, consumption of the pig head may alter intake of other minerals. It is important to note that if animals routinely receive enrichment items, other dietary items should be adjusted or formulated accordingly to account for nutrients coming from these items to prevent obesity, nutrient imbalance, or over nutrition.

Crude and total dietary fiber was low in pig heads. However, the fiber assays utilized were developed to capture plant-based fibers and likely do not accurately account for animal-based components. Therefore, the value of actual animal fiber in these items is likely underestimated. Recently, the concept of animal fiber (hair, bones, cartilage, etc.) has received more attention. Cheetahs consuming animal fiber in the form of whole prey, produced fecal phenol and indole concentrations that were 65.5 and 61.4% lower compared to cheetahs consuming ground raw meat diets, indicating a potential improvement in gut health [54]. Additionally, fiber can function as a pre-biotic, appetite regulator, and produce valuable short-chain fatty acids that provide energy for the large intestine [55]. An assay specific for determining non-digestible animal components would more accurately reflect the fiber present in animal-based enrichment items such as the pig head utilized in the current study. Evaluation of animal-based fiber in carnivore diets and enrichment items should be researched further as this assay currently does not exist.

### Nutrient intake from pig heads

Calculations of nutrient intakes can be used to adjust regular diet volumes based on degree of enrichment item consumption by an animal. Nutrient intake from consuming the heads was highly variable. Averages are presented to provide estimates of consumption and intake, but it is also important to look at minimum and maximum intakes per individual animal. In the current study, there was nearly a 250 g difference in minimum and maximum protein intake and more than a 2500 kcal difference in GE intake. For this reason, it is important to evaluate the loss of weight (i.e. consumption) in enrichment items, and perhaps preferential consumption of certain parts of enrichment items (i.e. fat, bone, eyes), for each animal individually because one animal may be consuming 3000 kcals from an enrichment item, while another may only consume 500 kcals. Considering individual ranges also may be important with other nutrients such as calcium and phosphorus. Additionally, future evaluation of nutrient composition of individual head components will allow more accurate estimations of nutrient intake associated with pig head consumption.

Macronutrient and energy digestibility was not evaluated in this study so gross energy can provide respective references. If calculated metabolizable energy (ME) is of interest, Atwater and modified Atwater factors could be used to predict estimates of ME. Modified Atwater values are typically used in ME calculations of commercial pet foods, however, unmodified Atwater factors have been shown to be more accurate in the ME calculations of raw meat diets [56,57]. Thus, ME calculations for this study were calculated using unmodified Atwater values as the pig head was raw.

When providing edible enrichment, nutritional contribution should always be considered. Presenting EE or diet in various forms is desirable but should not provide excess calories or nutrients to an animal. To do this, enrichment must be analyzed for nutrient composition and intake must be documented. Integration of nutrition and behavior when utilizing edible enrichment can optimize welfare and avoid undesirable metabolic outcomes.

## Conclusions

In conclusion, offering of a pig head as EE increased large cat activity compared to inedible enrichment items and increased activity was sustained 24 hrs post pig head offering (Post Enrichment). Novelty of the pig head also was maintained over a four-week period when offered once a week. Diet and fecal scores were not negatively affected by consumption of the pig head and no clinical signs were observed in any cats involved in this study. Ingestion of a pig head as EE may be variable and should be accurately accounted for in relation to overall species nutritional management. Future work should assess direct influence of temperature and RH on cat activity and diet consumption.

## Supporting information

S1 File Percent of observed time spent engaging in active and inactive behaviors by cats offered a pig head at Omaha’s Henry Doorly Zoo and Aquarium and Blank Park Zoo from June 30 to July 23, 2014. (XLSX)

S2 File Percent of observed time spent engaging in individual behaviors by cats offered a pig head at Omaha’s Henry Doorly Zoo and Aquarium and Blank Park Zoo from June 30 to July 23, 2014. (XLSX)

S3 File Number of exhibit location changes made by cats offered a pig head at Omaha’s Henry Doorly Zoo and Aquarium and Blank Park Zoo from June 30 to July 23, 2014. (XLSX)

S4 File Fecal scores of cats offered a pig head at Omaha’s Henry Doorly Zoo and Aquarium and Blank Park Zoo from June 30 to July 23, 2014. (XLSX)

S5 File Orts of cats offered a pig head at Omaha’s Henry Doorly Zoo and Aquarium and Blank Park Zoo from June 30 to July 23, 2014. (XLSX)

S6 File Time for cats to approach a pig head at Omaha’s Henry Doorly Zoo and Aquarium and Blank Park Zoo from June 30 to July 23, 2014 and loss of pig head weight. (XLSX)

S7 File Nutrient intake from consumption of pig head by cats at Omaha’s Henry Doorly Zoo and Aquarium and Blank Park Zoo from June 30 to July 23, 2014. (XLSX)
